# Developing consensus to enhance perinatal mental health through a model of integrated care: Delphi study

**DOI:** 10.1371/journal.pone.0303012

**Published:** 2024-05-09

**Authors:** Christine Ou, Zachary Daly, Michelle Carter, Wendy A. Hall, Enav Z. Zusman, Angela Russolillo, Sheila Duffy, Emily Jenkins

**Affiliations:** 1 School of Nursing, University of Victoria, Victoria, Canada; 2 School of Nursing, University of British Columbia, Vancouver, Canada; 3 Wellstream: The Canadian Centre for Innovation in Child and Youth Mental Health and Substance Use, Vancouver, Canada; 4 St. Paul’s Hospital, Providence Healthcare, Vancouver, Canada; 5 Department of Obstetrics & Gynaecology, University of British Columbia, Vancouver, BC, Canada; 6 Faculty of Health Sciences, Simon Fraser University, Burnaby, BC, Canada; 7 Pacific Post Partum Support Society, Burnaby, BC, Canada; School of Nursing Sao Joao de Deus, Evora University, PORTUGAL

## Abstract

Perinatal mental illness is an important public health issue, with one in five birthing persons experiencing clinically significant symptoms of anxiety and/or depression during pregnancy or the postpartum period. The purpose of this study was to develop a consensus-based model of integrated perinatal mental health care to enhance service delivery and improve parent and family outcomes. We conducted a three-round Delphi study using online surveys to reach consensus (≥75% agreement) on key domains and indicators of integrated perinatal mental health care. We invited modifications to indicators and domains during each round and shared a summary of results with participants following rounds one and two. Descriptive statistics were generated for quantitative data and a thematic analysis of qualitative data was undertaken. Study participants included professional experts in perinatal mental health (e.g., clinicians, researchers) (n = 36) and people with lived experience of perinatal mental illness within the past 5 years from across Canada (e.g., patients, family members) (n = 11). Consensus was reached and all nine domains of the proposed model for integrated perinatal mental health care were retained. Qualitative results informed the modification of indicators and development of an additional domain and indicators capturing the need for antiracist, culturally safe care. The development of an integrated model of perinatal mental health benefitted from diverse expertise to guide the focus of included domains and indicators. Engaging in a consensus-building process helps to create the conditions for change within health services.

## Introduction

Perinatal mental illness (PMI), which is mental illness experienced between the time of conception and the first year following childbirth, is an important public health issue. One in five birthing persons experience clinically significant symptoms of depression and/or anxiety during pregnancy or after giving birth [1–3] and an estimated one in 12 people report suicidal ideation [4]. Access to comprehensive and effective perinatal mental health (PMH) care is increasingly recognized as critical to improving long-term health and psychosocial outcomes for parents and their children [5–7].

Without access to holistic and coordinated PMH care, parents and children may experience profound health consequences. PMI is linked to adverse obstetric outcomes, such as preterm birth, low birth weight, small-for-gestational-age, stillbirth, and the need for operative deliveries [8,9]. PMI is also associated with adverse child outcomes, including developmental delays and socioemotional and behavioral challenges, such as attention deficit hyperactivity disorder [10–12]. Left untreated, PMI can become chronic, with widespread economic and social consequences [13,14]. Indeed, untreated or under-treated PMI is estimated to contribute substantial lifetime economic costs related to health and social care needs, and productivity and health-related quality of life losses [15,16]. In the US, untreated parental PMIs from 2017 alone were estimated to cost the system $14 billion from conception to 5 years after birth [17].

Internationally, screening and treatment for PMI is widely recommended, yet it remains unevenly executed [18]. Inconsistency in implementation results in a significant healthcare burden, including psychiatric admissions, outpatient treatment, and primary care visits [19]. In the Canadian context, access to clinically appropriate PMH care is hindered by the absence of routine screening for PMI and inadequately trained health care providers [20]. Even when symptoms are identified, systemic barriers to care prevent many individuals from receiving evidence-based treatment [21].

Despite high rates of PMI, the healthcare system is not equipped to adequately address care needs. A recent survey of perinatal care providers in Canada (n = 435) found that 96% of respondents believed that current PMH care services were insufficient to meet the needs of parents [20]. Moreover, the majority of care providers (87%) reported that many parents encounter significant barriers to accessing perinatal services, including limited availability, out-of-pocket costs, and a failure to address cultural safety [20]. As such, PMH services remain uncoordinated, with access often determined by ‘postal code lottery’, with those from urban, affluent, and well-resourced communities faring better than those from historically marginalized communities [20,22]. Because available services are often narrow in focus and ignore the broader social determinants of health—such as income and housing—pathways to screening, treatment, and follow-up tend to be unclear and difficult to navigate [20,23]. In the existing system, white, higher-income women with mild-to-moderate depression or anxiety often have better access to care and resources [22]. Parents who belong to historically underrepresented or marginalized groups, including rural residents, or those who are racialized, 2SLGBTQIA+, and/or living with complex mental illnesses, are particularly vulnerable to unmet PMH care needs [24,25].

Researchers and health professionals have argued that uncoordinated PMH services can be remedied by implementing an integrated model of PMH care. Integrated PMH care is the provision of coordinated, multidisciplinary care services—guided by evidence—to comprehensively address health and social care needs [5]. An integrated PMH care model can reduce system gaps and prevent the ‘screening paradox,’ wherein PMI is identified but left unmanaged because of a lack of referral pathways or resources [26]. An integrated PMH care model often uses a stepped care approach. That is, the least resource intensive care is offered first, ‘stepping up’ to progressively specialized, intensive PMH services as clinically indicated. Such a model is intended to make interdisciplinary and multimodal services available (i.e., coordinated medical, psychological, and pharmacological interventions) [6]. Primary care providers, many of whom do not have formal training in PMH care, can provide referrals, collaborate with PMH specialists, and access treatment records to support care trajectories [27,28]. Integrated PMH care is regarded as a health systems innovation that enhances perinatal mental health care equity by decreasing barriers to access and supporting the provision of coordinated care for all who need it based on best available evidence [28].

While integrated models of PMH care are considered promising for improving responses to population PMH care needs, there remains limited agreement on core model elements [19,29]. To address these knowledge and system gaps, this study aimed to generate a consensus-based model of integrated PMH care, with the potential to be applied in the Canadian health care context and beyond. Following, we detail our study using Spranger et al.’s reporting guidelines for Delphi techniques in health sciences [30].

### Study context

The present study was conducted in British Columbia (BC), Canada; a province that operates in the context of a publicly-funded health care system. This system was developed to provide citizens with medically necessary hospital and physician services without out-of-pocket costs. However, the system, as organized, has been referred to as a two-tiered system when it comes to mental health care (i.e., many mental health services are not publicly-funded).

Each province/territory is responsible for structuring and maintaining a system of health care for its residents. Although BC is recognized as a Canadian leader in advancing PMH care [23], services remain siloed and there are barriers to care that include inadequate screening, uneven resource distribution, and long wait times [31]. There is an urgent need for research to inform new models of care to enhance integration of existing services.

## Materials and methods

### Study design

This study used a three-round Delphi design to inform a consensus-based model of integrated PMH care. To ensure the consideration of diverse perspectives, panel members included health care providers, researchers, and people with lived experience of PMI. Delphi methods use multi-stage panel surveys, which can include qualitative and quantitative data, to facilitate consensus building among experts [31]. Following each stage of surveying, feedback is provided to panel participants (e.g., aggregated survey results in the form of a short report). Panel participants are then provided with the opportunity to reconsider their responses in relation to this feedback and to indicate their evolving perspectives through subsequent survey rounds [31,32].

Delphi items can be generated through empirical evidence in combination with expert perspectives [33]. To identify core, evidence-informed domains (i.e., dimensions of integrated perinatal mental health care) and indicators (i.e., attributes of each dimension) [33], our study began with a scoping review of empirical literature (manuscript in-preparation). To populate a Delphi consensus-building survey, our diverse and interdisciplinary research team, which includes people with lived experience of PMI, used evidence from this scoping review to identify domains and indicators relevant to an integrated model of PMH care.

### Participant identification and recruitment

Panel participants were recruited from across Canada and included perinatal clinicians and service providers (e.g., nurses, midwives, physicians, doulas, psychologists, pharmacists, social workers), researchers, and PMI patients and family members. Professional experts (i.e., clinicians, researchers, service providers) were eligible to participate if they had a minimum of two years of experience working in the context of PMH care. Patient and family experts were eligible to participate if they had experiences with PMI within the past five years. We identified a diverse group of subject experts resulting in an initial list of potential participants (n = 94). Prospective panel members were provided with the study purpose and invited to complete a series of confidential Delphi consensus-building surveys consisting of queries with pre-determined response options (i.e., quantitative) and open-ended questions (i.e., qualitative). Surveys were created in Qualtrics and a survey link was included in the invitation email, with prospective participants encouraged to share the invitation within their networks to facilitate recruitment. Ethics approval for this study was obtained from the University of British Columbia Behavioural Research Ethics Board (H21-00651). Participants completed an online consent form prior to initiating the survey and were provided with an honoraria of $25 CAD (gift card) in Rounds 1 and 2, and $50 CAD (gift card) in Round 3 to improve retention.

### Data collection

#### Delphi Round 1

The survey link was emailed to prospective participants in October 2021. Participants were asked to evaluate the indicators in each identified domain for relevance (i.e., whether the indicator contributes to an integrated model of PMH care), importance (i.e., the value of the indicator to an integrated model of PMH care), and relative rank (i.e., the comparative importance of the indicator relative to other indicators). Comments and suggested modifications for the proposed domains and indicators were also invited. Participants were asked to assess the relevance of each indicator with the response options: “Keep,” “Modify,” or “Remove.” Participants were asked to assess importance with the response options: “Not at all important,” “Somewhat important,” “Very important,” and “Extremely important.” Participants were asked to rank indicators in the order of greatest to least importance. Based on common practice in Delphi surveying, a threshold of ≥ 75% agreement across participants was considered to indicate consensus on the relevance and importance of assessed indicators [34]. We also sought open-ended feedback on the domains and indicators, as well as input on content that participants perceived as missing from the model. The first round of surveying included 44 indicators across nine domains, and our primary goal was to assess consensus for retaining the proposed indicators. Participants were given a 2-week window to submit the survey and received reminders to encourage completion.

#### Delphi Round 2

Following Round 1 surveying, we summarized the results, including qualitative feedback, and provided a summary report to all participants. In December 2021, a second survey was emailed to participants who were asked to re-assess each domain and indicator while taking into consideration other participants’ input. Our primary goal for this round was to collect additional qualitative data to inform potential modifications. All participants from Round 1 were invited and sent weekly reminders to complete the survey within the two-week timeframe.

#### Delphi Round 3

Following Round 2 surveying, we summarized the results and provided a summary report to all participants. In response to Round 2 findings, which identified the need for another domain and additional indicators, a third round of surveying was initiated in April 2022. Based on conversations with study partners who work in health services administration, we invited 16 additional participants to Round 3 to bring further diversity, specifically the perspectives of Indigenous and 2SLGBTQIA+ people. Participants in Round 3 were again asked to provide information on the relevance, importance, and relative rank of the modified and retained items as well as the added domain and related indicators. Comments about further potential modifications and general perceptions were invited. All participants from Round 2 as well as the new Round 3 participants were sent weekly reminders to complete the survey within the two-week timeframe.

### Data analysis

All quantitative data were uploaded to SPSS version 28.0 to facilitate analysis. Descriptive statistics were used to characterize the sample and to assess consensus on the relevance and importance of domains and indicators across survey rounds. To examine relative rank, we drew on Round 3 data and used the Borda Count method to produce a summary score. The Borda Count is a scoring method where indicators are awarded points according to their rankings [35]. Where participants skipped the ranking process within a domain, they were excluded from analysis. If participants partially completed the ranking (e.g., only assigned ranks to their top three indicators), the unranked indicators were allotted a score of one [35]. Qualitative data were analyzed according to survey round, domain of interest, and indicator examined. Using an inductive approach, data were clustered into subthemes and higher order themes, establishing links between participant responses.

## Results

### Participant overview

Of the 94 prospective participants invited, 31 participated in Round 1. Of these, a total of 20 returned to participate in Round 2. In Round 3, 13 participants returned from Round 2 and two participants returned from Round 1. Additionally, 16 new participants were recruited for a total of 31 Round 3 participants. Across all three rounds, the majority of participants identified as women/female, lived in a metropolitan area, and had attended/completed post-secondary education. The majority of participants were clinicians or direct care providers, with approximately half of participants residing in British Columbia. The proportion of participants who were PMH care service users (i.e., patients or family members) ranged from 16% in Round 1 to 29% in Round 3 (see Table 1).

**Table 1:** Sociodemographic characteristics across all three rounds.

	Round 1 (N = 31)		Round 2 (N = 20)		Round 3 (N = 31)	
	n	%	n	%	n	%
**Gender/Sex**						
Woman/Female	30	96.8	19	95.0	29	93.5
Man/Male	0	0.0	0	0.0	0	0.0
Non-binary	1	3.2	1	5.0	1	3.2
Transgender	0	0.0	0	0.0	1	3.2
**Ethnicity** *						
Indigenous	2	6.5	2	10.0	2	6.5
Racialized	5	16.1	3	15.0	8	25.8
Non-Racialized	24	77.4	15	75.0	21	67.7
**Region**						
British Columbia	13	41.9	10	50.0	17	54.8
Atlantic Canada	3	9.7	1	5.0	1	3.2
Ontario	5	16.1	5	25.0	6	19.4
Prairies	6	19.4	2	10.0	4	12.9
Quebec	4	12.9	2	10.0	3	9.7
**Setting**						
Metropolitan/Major City	20	64.5	12	60.0	22	71.0
Regional/Urban/Large Town/Small City	8	25.8	6	30.0	9	29.0
Rural/Small Town	3	9.7	2	10.0	0	0.0
**Highest Level of Completed Education**						
High school	1	3.2	0	0.0	1	3.2
Bachelor’s Degree/College/Technical School	7	22.6	5	25.0	6	19.4
Master’s Degree/Doctorate/Professional Degree	23	74.2	15	75.0	24	77.4
**Role**						
Perinatal mental health service user (family member)	0	0.0	0	0.0	1	3.2
Perinatal mental health service user (patient)	5	16.1	4	20.0	8	25.8
Clinician/Care provider	16	51.6	10	50.0	14	45.2
Researcher	10	32.3	6	30.0	8	25.8

*Participants were asked to report their cultural/ethnic heritage. Those who reported solely European origins were considered non-racialized, those who reported one or more non-European, non-Indigenous origins were considered racialized, and those who reported Indigenous origins were classified as Indigenous.

### Quantitative results

Ratings of relevance and importance are provided in Table 2. Consensus was reached for the relevance of all indicators within each domain across all three rounds (i.e., 44 indicators across 9 domains in Rounds 1 and 2, and 57 indicators across 10 domains in Round 3). The number of indicators reaching consensus (≥ 75% agreement) as being “extremely important” or “very important” to an integrated model of PMH care varied across rounds: 43 of 44 indicators reached concensus in Round 1, 37 of 44 indicators reached consensus in Round 2, and 53 of 57 indicators reached concensus in Round 3. The four indicators that did not achieve consensus as being “extremely important” or “very important” in the third and final round were: indicators 4 and 6 in the “Person-centred care” domain; indicator 5 in the “Transition and discharge planning” domain; and indicator 3 in the “Care model planning” domain (see Table 3). Furthermore, in Round 3, 4 of 57 indicators achieved consensus on being “extremely important”. These were indicators 3 and 5 in the “Health promotion and illness prevention” domain, and indicators 3 and 5 in the “Biopsychosocial approach to treatment” domain (see Table 3). The relative rankings of indicators using Borda count methods are provided in Table 2. None of the four indicators that reached consensus on being extremely important were the highest ranked in their respective domains. Indicators that failed to reach consensus on importance were also the lowest ranked in their respective domains (see Table 2).

**Table 2:** Proportion of indicators achieving consensus* for relevance and importance across all three rounds.

Domain name	Number of Indicators Per Domain			Proportion of Indicators for which Consensus was Reached on Relevance (i.e., to “Keep” the indicator)			Proportion of Indicators for which Consensus was Reached on Importance (i.e., "Extremely important" or "very important")			Proportion of Indicators for which Consensus was Reached on Importance (i.e., "Extremely important" only)		
	Round 1	Round 2	Round 3	Round 1	Round 2	Round 3	Round 1	Round 2	Round 3	Round 1	Round 2	Round 3
**Person-centred care** **	4	4	7	100%	100%	100%	100%	75%	71%	50%	50%	0%
**Cultural safety, cultural humility, & anti-racism** ***	---	---	8	---	---	100%	---	---	100%	---	---	0%
**Care delivery**	5	5	5	100%	100%	100%	100%	80%	100%	0%	0%	0%
**Health promotion & illness prevention**	5	5	5	100%	100%	100%	100%	100%	100%	40%	20%	40%
**Screening, assessment, & triage**	5	5	5	100%	100%	100%	100%	100%	100%	20%	20%	0%
**Biopsychosocial approach to treatment**	5	5	5	100%	100%	100%	100%	80%	100%	0%	0%	40%
**Transition & discharge teaching**	6	6	6	100%	100%	100%	83%	67%	83%	33%	0%	0%
**Training & education**	4	4	4	100%	100%	100%	100%	100%	100%	25%	25%	0%
**Care model planning**	6	6	8	100%	100%	100%	100%	67%	88%	17%	0%	0%
**Care model evaluation**	4	4	4	100%	100%	100%	100%	100%	100%	0%	25%	0%
**Total:**	44	44	57	100%	100%	100%	98%	84%	93%	20%	14%	7%

* Defined as ≥ 75% of respondents selecting the same response for an indicator (for relevance or importance).

** This domain was named “Patient-centredness” in Rounds 1 and 2, but was renamed as part of other modifications in Round 3.

*** This domain was added in Round 3.

**Table 3:** Consensus-based model of integrated perinatal mental health care domains & indicators.

Domains and Indicators	Round 3 Relative Rank^§^
** 1. Person-Centred Care**	
*Domain description*: This domain incorporates an intersectional understanding of PMH clients, recognizing each person’s unique preferences, values, and circumstances in decision-making, and the ways in which they may experience oppression within and beyond health services.	
*Indicators*:	
1. PMH clients are an integral part of decision-making processes, in collaboration with the care team.	1
2. The care team shares information with PMH clients clearly, fully, and in a timely manner to support informed decision-making.	2
3. PMH clients are supported by the care team to develop individualized goals of care that reflect personal preferences, values, cultural traditions, and life circumstances.	3
4. The care team facilitates involvement of (client-identified) family/advocates in PMH care settings.	6
5. The care team acknowledges that PMH inequities (e.g., access to care, quality of care, health outcomes)–rooted in power asymmetry and systemic hierarchies–are shaped by the interaction of multiple overlapping social factors such as race, income, education, age, ability, sexual orientation, immigration status, ethnicity, and geography.	4
6. The care team practices ongoing critical reflection to examine social determinants of health, issues of equity, processes of stigmatization, experiences of oppression, and the operation of power in PMH policy-making, care delivery, and resource allocation. *	7
7. The care team seeks to address PMH disparities through policies and programs that enhance health equity and integrate gender-affirming, anti-discrimination, and human rights-based approaches. *	5
** 2. Cultural Safety, Cultural Humility, & Anti-Racism**	
*Domain description*: This domain focuses on culturally safe care, an outcome based on respectful engagement that recognizes and addresses power imbalances in the health care system. Cultural safety results in an environment free of racism and discrimination, where people feel safe when engaging with health care professionals and receiving health care.	
*Indicators*:	
1. The care team practices cultural humility, a process of life-long self-reflection to examine personal and systemic biases, assumptions, and power imbalances that can serve to restrict cultural norms or values of Black, Indigenous, and People of Colour (BIPOC) and create harm. *	1
2. The care team acknowledges the history of racism in Canada and the effects of systemic racism on individual and population health, wellness, and health care experiences. *	6
3. The team commits to, and enacts, the Truth and Reconciliation Commission of Canada Calls to Action. *	7
4. The care team seeks to improve their provision of culturally safe care by undertaking ongoing education on BIPOC health care, determinants of health, cultural safety, cultural humility, and anti-racism. *	4
5. The care team creates and maintains physical spaces that are culturally safe, welcoming, and connected with other culturally safe services. *	2
6. The care team engages in culturally appropriate and respectful communication by reducing language barriers and avoiding communication that disempowers, humiliates, or excludes people. *	3
7. The care team engages in meaningful partnerships with BIPOC in the planning and delivery of culturally-safe PMH services, integrating traditional cultural practices into individualized care that meets the health care needs of individuals and families. *	5
8. Cultural safety is continually assessed by the systematic monitoring and evaluation of inequities in care experiences and health outcomes. *	8
** 3. Care Delivery**	
*Domain description*: This domain describes the design and delivery of integrated care systems that provide interdisciplinary PMH services to improve care quality, care continuity, client experience, and health outcomes.	
*Indicators*:	
1. Team Based Care: The care team is interdisciplinary, with defined competencies, roles, responsibilities, and boundaries to deliver a comprehensive continuum of PMH care.	1
2. Communication: In concert with clients, protocols are developed for specialty referrals and communication across the care continuum.	4
3. Stepped Care: The most effective, least resource intensive, care is offered first, ‘stepping up’ to progressively specialized, intensive PMH services as clinically indicated.	3
4. Integrated Care: Processes are in place to ensure coordinated movement from one care team (or level of care) to another.	2
5. Shared Information Systems: Information systems (e.g., health records) and decision support tools (e.g., clinical protocols) are structured to promote data sharing, care coordination, and collaborative decision-making among clients, approved family/ support people, and other PMH team members.	5
** 4. Health Promotion & Illness Prevention**	
*Domain description*: This domain describes strategies to enhance individuals’ agency to increase control over their PMH (health promotion) and prevent PMI (illness prevention).	
*Indicators*:	
1. PMH education is provided to all pregnant and postpartum persons and their support people.	1
2. PMH education includes information about PMI prevalence, risk factors, and symptoms; potential impacts of untreated PMI on family and child health; strategies to promote mental health; and access to community PMH resources and/or cultural support services.	4
3. Individuals who have pre-existing mental illness(es) and planning a pregnancy receive timely preconception PMH care.	2
4. Preconception PMH care includes facilitating clients to optimize health behaviours (e.g., nutrition, exercise, sleep, time for self), respecting risky behaviours, providing harm reduction resources, mobilizing social and structural support (e.g., food or housing resources), identifying PMI risk factors/individual triggers (including intergenerational trauma)/early signs or recurrence, and offering a client directed approach to treatment decision-making.	5
5. When an individual with mental illness(es) becomes pregnant, the client and care team co-develop an individualized plan to address mental health care, social circumstances, early signs of illness recurrence, crisis management, prenatal care, labour management, postpartum care, and infant care.	3
** 5. Screening, Assessment & Triage**	
*Domain description*: This domain focuses on processes related to identifying the risk or possible presence of PMI (screening), characterizing PMI to inform a diagnosis and care plan (assessment), and evaluating the nature and severity of PMI to determine type and timing of services (triage).	
*Indicators*:	
1. All pregnant and postpartum people are routinely screened for PMI using a validated tool (e.g., Edinburgh Perinatal Depression Screen, Patient Health Questionnaire-9).	1
2. A positive screen includes endorsed thoughts of self-harm or suicide and/or a total score above the cut-off value associated with a validated tool.	5
3. PMI screening ideally occurs in care settings that include PMH-educated clinicians, social support, and community resources for families, and client choice or self-referral protocol for follow-up when screening is positive.	3
4. Screening follow-up includes a comprehensive PMH assessment used to inform a possible diagnosis, identify safety risks (e.g., intimate partner violence), and the development of an individualized care plan, developed in partnership with the client.	2
5. The care team reflects on the nature and severity of symptoms, client preferences, and social circumstances to inform the type and timing of PMH services and supports (including traditional knowledge and teaching) offered.	4
** 6. Biopsychosocial Approach to Treatment**	
*Domain description*: This domain describes evidence-based treatment strategies that incorporate client preferences, as well as the physical, psychological, and social aspects of PMI, with a focus on symptom reduction and relapse prevention.	
*Indicators*:	
1. Treatment is offered within the context of an integrated, stepped-care model, ensuring intervention intensity matches client need and care is coordinated across clinical teams and care settings.	1
2. A treatment plan specifies a range of intervention options adaptable to clients’ needs and preferences.	3
3. Evidence-based interventions offered for PMI include psychoeducation, psychotherapy (e.g., cognitive behavioural therapy, interpersonal therapy, parent-infant psychotherapy), traditional healing approaches, and medication.	4
4. The treatment plan incorporates culturally-safe and trauma-informed solutions for mental health risk factors and symptoms, obstetrical/medical risk factors and symptoms, psychosocial needs, child care support, and community resources.	2
5. Ongoing assessment and monitoring focus on treatment effectiveness and potential treatment modifications.	5
** 7. Transition & Discharge Planning**	
*Domain description*: This domain refers to the coordination, communication, and resources required to effectively support PMH clients move between health care settings and into the community.	
*Indicators*:	
1. Care transitions are guided by clear, written communication of care plans between care teams.	4
2. Care transitions address client goals, interventions and treatment, client/infant safety planning, and social needs.	1
3. Transitions such as discharge planning are guided by the client in collaboration with the health care team, (client-identified) family/advocates, and community supports.	2
4. Transition and discharge plans are aligned with the clients’ goals and preferences.	3
5. Transition planning begins when a client engages in care with a PMH clinician or care team.	6
6. Transitions incorporate care team awareness of trauma-informed and culturally sensitive PMH community resources and client opportunities to access appropriate services and culturally-relevant programs.	5
** 8. Training & Education**	
*Domain description*: This domain focuses on education and training to enhance the skills and competencies (including cultural competency) of clinicians and support people working with PMH clients.	
*Indicators*:	
1. PMH education focuses on building capacity to acknowledge, respect, and integrate clients’ past experiences, cultural beliefs, and personal values (e.g., trauma-informed care, Indigenous cultural safety, harm reduction) into care.	2
2. Core PMH training competencies include application of (1) biological and psychosocial/cultural underpinnings of perinatal transitions; (2) risk factors and spectra of mental illness(es) across the perinatal period, appropriate screenings, assessments, and interventions; and (3) application of PMI treatment approaches (e.g., psychotherapy, medications, inpatient care, social referrals) that acknowledge systemic racism and incorporate parental, infant, and family wellbeing.	1
3. On-the-job performance support (e.g., clinical supervision, peer mentoring, team huddles, client feedback, and written resources) to reinforce and strengthen PMH training and competencies.	3
4. Plan for PMH education revisions to ensure current culturally safe and trauma-informed content and team support.	4
** 9. Care Model Planning**	
*Domain description*: This domain describes the relationship between people, processes, and systems needed to prepare an organization for implementation of a culturally-safe, trauma-informed, integrated PMH model of care.	
*Indicators*:	
1. The planning team conducts a local needs assessment to identify systemic racism, and gaps and needs in PMH services.	4
2. The planning team assesses care model feasibility and acceptability through stakeholder engagement (emphasizing client perspectives).	1
3. The planning team identifies guiding principles underpinning aims and objectives to support decision-making.	7
4. The planning team defines the nature of clients, scope and relevance of clinical services, and team composition (including roles and responsibilities) for the care model.	2
5. The planning team identifies space and resource requirements to support a culturally-safe and trauma-informed PMH care model.	5
6. The planning team identifies systemic barriers to accessing PMH services (e.g., stigma, awareness of services, language/cultural barriers, child care, etc.).	3
7. In concert with communities served and clients, the planning team develops resources to reduce barriers, including funding when specialty services require client relocation. *	6
8. The planning team develops materials to support care model operationalization (e.g., work flow diagrams, communication forums, protocols, educational materials, etc.), with a planned schedule for updating supporting materials. *	8
** 10. Care Model Evaluation**	
*Domain description*: This domain focuses on the evaluation of care models to facilitate continuous learning and improvement.	
*Indicators*:	
1. An evaluation plan specifies the purpose of the evaluation, key evaluation questions, and key indicators.	1
2. An evaluation plan indicates how data will be collected, analyzed and reported.	4
3. Evaluation questions include systemic features influencing care model access and utilization, cultural safety associated with clinical service delivery/treatment options, and clients’ experiences/outcomes.	2
4. Results identify effective care model practices, indicators of barriers/problems, and areas for quality improvement.	3

PMH: perinatal mental health; PMI: perinatal mental illness.

^**§**^ Relative rank was derived using Borda Count method.

* This indicator was added in Round 3.

### Qualitative results

Although the quantitative data indicated a high level of agreement on the relevance, and to a lesser extent the importance of indicators, qualitative data from the open-ended responses provided a more nuanced picture. In total, four themes were developed from the qualitative data: representation of care recipients; care recipient advocacy; language for cultural safety, cultural humility, and anti-racism; and incorporation of systemic approaches.

Participants who responded with modifications for indicators and suggested additional indicators, emphasized the importance of attention to representation of care recipients (Theme 1). There were two subthemes. The first was rejection of the term ‘patient’. Participants who objected to using patient as a descriptor offered alternatives, such as persons, people, or clients. For example, “people rather than patients” was a sentiment provided frequently in Round 1. The second subtheme was family inclusion. Participants consistently commented about including family, as defined by the care recipient, in the indicators for domains, including person-centred care, care delivery, health promotion and prevention, biopsychosocial approach to treatment, and transition and discharge planning. This included comments like, “Health promotion activities with the whole family unit” (Round 1) and “I think preconception planning could also include relationship support and co-parenting plans, since this seems to be associated with people staying well over time.” (Round 2).

The second theme suggested indicator modifications and additional indicators related to care recipient advocacy. In all three rounds, participants emphasized the importance of starting from the perspective of the care recipient and care recipients advocating for themselves. They argued that adequate information about options for care was fundamental to care recipients’ efforts to collaborate with care providers by taking the lead and expressing preferences (choices). For example, “As a patient, I didn’t know what to expect. Just being asked "what do you need?" isn’t always enough when we aren’t necessarily aware of what options exist. I think more guidance around what is doable within the patient’s goals (Round 1)”. There were two subthemes: providing options to support collaboration and supporting care recipients’ choices. Providing options to support collaboration, represented a number of comments about the importance of information. For example, “if the client doesn’t know options, unable to lead” (Round 1) and “takes the lead after an informed choice discussion” (Round 2). In the supporting choices subtheme, participants wrote how indicators could be altered. For example, “all patients are offered routine screening–need to ensure patient autonomy and choice to be screened” (Round 3) and “providing an option for self-referral or self-advocacy for clients…” (Round 1).

The third theme was language for cultural safety, cultural humility, and anti-racism. Many participants responded to the new domain, identified in Round 3. Participants had consistently written about the need for attention to cultural safety in Rounds 1 and 2. For example, “the care team collaborates with patients and their families to develop individualized care plans that are trauma-informed and culturally sensitive” (Round 1) and “in a culturally safe way” (Round 2). Participants paid close attention to Round 3 indicator language. There were three subthemes: White supremacy, objections to BIPOC, and inadequate team power. The first subtheme was supported by lengthy comments. Participants indicated that White supremacy and ‘current’ racism in Canada required acknowledgement. For example, “the care team practices cultural humility, a process of life-long self-reflection to examine personal and systemic biases, assumptions, and power imbalances that serve to maintain the status quo of White supremacy and marginalization of Black, Indigenous, and People of Colour (BIPOC).” Some of the participants objected to the use of BIPOC. They regarded the term as limiting and suggested that it excluded marginalized groups, such as immigrant populations. For example, “Indigenous first, avoid acronyms as they can change and are limiting.” The third subtheme was inadequate team power. Some participants expressed concerns that care teams did not have the wherewithal to make the changes spelled out in the indicators. For example, with regards to culturally safe services, one participant wrote, “I don’t think you can put this on the care team. It may not be up to them. I think that you need a different level of indicators for the people in charge.” With regards to calls to action from the Truth and Reconcilliation Commission of Canada, which sought to reflect the stories of those who were impacted directly or indirectly by the Indian Residential Schools system in Canada, another participant wrote, “The care team might not have that power”.

The fourth and final theme was: incorporation of systemic approaches. Some participants expressed concern about the model operating at an ‘individual’ level. For example, participants described indicators that could capture the importance of support and social determinants of health in health promotion: “Place weight on in-home and community supports, socio-economic status, access to other resources as a factor in triage beyond individual mental health indicators” (Round 1).

An important subtheme was the universality of screening for PMIs. Participants objected to limiting screening to particular settings and groups. For example, “I think the word ‘universal’ must be included in screening, it can be done in multiple settings, [Canada Prenatal Nutrition Program] and other maternity programs, on pregnancy, postpartum and lactation apps…” (Round 1) and “Screening may happen in settings with a trained health care professional who is aware of next steps, have had education and are aware of resources in health care and community” (Round 2).

In the second subtheme, participants wanted treatment and transition plans to attend to social determinants of health, barriers to treatment, and stakeholder engagement. They were concerned about silos rather than integration of community and acute care settings. For example, “Ongoing assessment and monitoring includes: treatment effectiveness and potential treatment modifications, changes in social determinants of health for the client and approaches to meeting fundamental needs…, psychosocial and relational risk factors…, and collaborative problem-solving around child care support/psychosocial needs” (Round 3) and “The treatment plan is trauma-informed, culturally sensitive, and responsive to patients’ values, preferences and needs…” (Round 1).

In the third subtheme, the participants called for national components to support a perinatal mental health model. They wanted standardization. For example, “National infrastructure developed as a central resource for up-to-date educational offerings and maintaining/curating educational resources (a "one-stop shop" for those working in PMH care to stay current)” (Round 1).

In general, comments about the model indicated a preference for more continuity of care from various settings; separating out each treatment component and changing the weighting of indicators to actual treatment choices; simplifying the language; and reducing the clinical focus of the model. Participants’ acknowledged leadership in thought and time devoted to the survey; attention to trauma-informed care; intersectionality with BIPOC and Indigenous issues; and attention to culturally competent care.

The themes and general comments provided context for the changes made in indicators and domains (e.g., new label for person-centered care instead of patient-centred care, new domain for cultural safety, cultural humility, and anti-racism) for Round 3 of the Delphi Survey.

#### Summary of changes to the model during the delphi process and resulting final version

In Rounds 1 and 2, the model presented to participants was identical. In response to participants’ comments in Rounds 1 and 2, modifications and additions were made to the model that was assessed in Round 3, including the addition of a new domain, new indicators, and modified indicators. The final version of the model, including all domains and indicators is presented in Table 3.

## Discussion

There is growing recognition of the need to improve accessibility and coordination of evidence-based PMH care to enhance parent and family outcomes. Despite awareness of current gaps in services, the essential components of an integrated model of PMH care remain unclear. In this study, our interdisciplinary team proposed a model of integrated PMH care developed through a review of evidence designed to address this gap in knowledge. Using Delphi methods, we then sought input from a diverse group of stakeholders, including clinicians, researchers and people with lived experience of PMI in order to gain consensus on key model components. Implementing the resulting model may enhance the quality, accessibility, and effectiveness of PMH care services, thereby improving outcomes for parents and families.

While there are profound siloes that continue to serve as a barrier to good care and outcomes for patients navigating PMI, we achieved evidence-aligned consensus among experts and people with lived experience of PMIs for the key elements of an integrated model of PMH care. Research on integrated PMH care services has underscored the importance of coordinated and scaffolded services that feature inter-professional collaboration, shared vision and goals, continuity of care, and adequate funding and resources to support coordination [36,37]. Indeed, evidence from systematic reviews synthesizing research on non-perinatal integrated models of care indicate that they improve care quality and access, while also lowering operational costs [38] and improving patient satisfaction [38,39]. In the perinatal setting, preliminary evaluation of a state-wide integrated model of PMH care in the US demonstrated improvements in mental health outcomes and patient satisfaction [40]. Qualitative findings from our study extend these findings and indicate the need for centralized processes to support care coordination, referrals, and education. Indeed, adopting a national strategy to provide a coordinated approach to PMH care holds the potential to harmonize screening practices and care standards across provinces and territories, while also enhancing integration and access to care through centralized funding and resource allocation [20,22,23].

Methodologically, the Delphi process emphasized the need for careful consideration of data used to determine the acceptability and sufficiency of a proposed model of care. Indeed, while Delphi methods provide an approach to building consensus on issues of interest in health contexts, relying solely on the collection of quantitative data risks prematurely–and inaccurately–declaring consensus as having been achieved. In the context of this study, the quantitative data provided an incomplete picture of the adequacy and comprehensiveness of the proposed integrated model of PMH care. For example, consensus on the relevance of indicators was achieved in all three rounds of surveying, and the majority of indicators reached consensus in relation to importance. If the team relied exclusively on quantitative data, this study may have been considered complete after Round 1 surveying. Qualitative input suggested that several indicators required modification and that an additional domain was required. This feedback was carefully considered by the study team and informed the creation of the domain of “Cultural safety, cultural humility, and anti-racism”, as well as the alteration and addition of several indicators. Indeed, a mixed methods approach provided critical insights and new understandings that would not have been identified if the study relied solely on quantitative data. Qualitative data invites diverse perspectives and expertise to be shared to bring richer insights and support a collaborative process where panel participants can more meaningfully inform the model or framework generated [41,42]. The utility of collecting both qualitative and quantitative data in Delphi studies has been noted by others who have observed that a mixed methods approach is essential for identifying framework deficiencies and necessary modifications [41–43].

Beyond the importance of using mixed methods, this study benefited from attention to the characteristics and composition of the participant panel. For example, minority perspectives risk being overshadowed when relying on numerical consensus methods alone. As such, efforts were made to enhance participant heterogeneity through the purposive sampling of participants who identified as belonging to an equity-deserving group, such as recent immigrants and people who identify as 2SLGBTQIA+. While the attrition of participants from Round 1 and the introduction of new participants in Round 3 may have reduced consensus on the importance of certain indicators, inviting new participants to Round 3 facilitated a diversity of perspectives in assessing the new domain of “Cultural safety, cultural humility, and anti-racism” and related indicators. As noted by others, there is an inherent tension that needs to be managed between facilitating sample homogeneity to reach consensus and maintaining enough heterogeneity to detect model gaps [44].

The consensus-based model of integrated PMH care generated through this study provides key insights that build on the existing research in this field. Importantly, our panel participants highlighted the need for greater emphasis on culturally safe care in PMH programming. This omission was identified by several participants in Rounds 1 and 2 through qualitative feedback. The addition of the cultural safety, cultural humility, and anti-racism domain addresses a critical gap in much of the existing research on PMH care. This domain acknowledges that childbearing people from equity-deserving groups (e.g., racialized and Indigenous peoples) routinely encounter racism and discrimination in health care [45,46]. Racism and discrimination are embedded in organizational structures of health care in many Western contexts, resulting in inequities in maternal and infant morbidity and mortality among certain population groups [41,47,48]. Moreover, socioeconomic disparities often compound racial inequities [48,49]. For example, research has demonstrated that racialized women are less likely to be screened for depression [50] and to receive treatment [51]. The addition of this 10^th^ domain is aligned with recent efforts to recognize and redress issues of equity and justice in health care access and treatment for populations who experience structural vulnerabliity [52,53].

### Strengths & limitations

This study provides new insights and a consensus-based model of integrated PMH care informed by a geographically, ethnoculturally, and experientially diverse participant panel; however, there are limitations that warrant discussion. To present the various domains and indicators of an integrated model of PMH care, the Delphi survey was long and participation required a substantial time commitment. The time commitment likely contributed to attrition following the Round 1 survey. Efforts were made to expand the panel and more appropriately acknowledge contributions through a larger honoraria offered in Round 3. Data collection issues arising from the question structure on the Qualtrics platform precluded the use of ranking data from Rounds 1 and 2. That problem holds implications for insights into the relative importance of the indicators. We resolved the issue in Round 3, by providing an indication of relative importance of indicators in this final survey. Finally, while the qualitative data provided important findings that led to the creation of a new domain as well as related indicators, the collection of these data through open-ended survey questions limits the opportunity for more nuanced exploration or validation of interpretations. Future research would benefit from the incorporation of qualitative focus groups to further refine and finalize model components.

### Clinical implications

This study developed a consensus-based model of integrated PMH care that holds the potential to guide health services redesign to improve PMH care treatment, access and patient experience [54]. Future research should include pilot testing to assess the feasibility and acceptability of this model in diverse care contexts [55]. Data on process and outcome measures and qualitative feedback from service users and clinicians can inform model refinements to improve usability and effectiveness [54]. Model implementation feasibility metrics–such as cost-effectiveness and stakeholders’ willingness to adopt the model–have utility to determine the model’s practicality, scalability, and potential for wider application [54,55].

## Conclusion

Perinatal mental illness is a key public health issue requiring innovation in health services planning and delivery to improve outcomes for parents and their children across the life course. Using Delphi methods, this study contributed to of the development of a consensus-based, evidence-aligned and “expert”-informed model of integrated PMH care to guide health systems and practice change. Grounded in scientific evidence the model was strengthened by the incorporation of expert and lived experience knowledge to include 10 priority domains: person-centred care; cultural safety; care delivery; health promotion; screening; biopsychosocial treatment; transition support; education; strategic care model planning; and evaluation. Future research is needed to support implementation and testing of this model in diverse settings.
